# Undergraduate medical education: Psychological perspectives from India

**DOI:** 10.4103/0019-5545.37317

**Published:** 2007

**Authors:** L. S. S. Manickam, T. S. Sathyanarayana Rao

**Affiliations:** Department of Psychiatry, JSS Medical College Hospital, Mysore - 570 004, India

## INTRODUCTION

Undergraduate medical education in India in the new century is facing more challenges than ever before. Apart from the advanced technological input that came into health-care practices, the fast-changing socioeconomic cultural scenario is also posing a grave concern in the process of producing quality physicians to meet the demands of the future.

Though the number of medical colleges has increased substantially to meet the health-care needs of the country, the institutions have to compete with each other to get expert medical educationists who could impart effective training. Highly lucrative jobs that are offered to an engineering or IT professional after four years of less strenuous training shake up the medical student, as well as those who are aspiring to take up the medical profession, and make them ponder whether it is worth its effort in terms of job satisfaction and remuneration. Recent introduction of changes by the policy makers, including the compulsory service in rural areas by the newly trained doctors, could pose an added stress. This is complicated by the fact that even in rural settings, the patients, as well as their relatives, have become so increasingly better informed that they tend to question the decisions and approaches of the physicians to the extent of affecting the doctor-patient relationship. In this paper we review the present curriculum in terms of the psychology component and the probable ways in which it can be effectively implemented in order to train a physician who could be facing the challenges posed, and propose to review the current selection procedure to recruit those with good aptitude for the profession.

## THE PRESENT CURRICULUM AND THE LEARNING OBJECTIVES RELATED TO PSYCHOLOGY

The Kacker committee, constituted by the Medical Council of India (MCI),[1] recommended to include “humanities” in the pre-clinical phase and to examine the students in it. Accepting the suggestions, the curriculum provided by MCI[2] included knowledge content and skill related to psychology, under “psychiatry” and recommended that “Training should be integrated with the departments of Medicine, Neuroanatomy, Behavioral Sciences and Forensic Medicine.” At the knowledge level, it is stipulated that the student should be able to “comprehend nature and development of different aspects of normal human behavior like learning, memory, motivation, personality and intelligence” and “recognize differences between normal and abnormal behavior.” At the psychomotor or skill level, the student should be able to effectively:

1. Interview the patient and understand different methods of communication in patient-doctor relationship.
2. Identify and manage psychological reactions and psychiatric disorders in medical and surgical patients in clinical practice and in community setting.

However, only a small minority of institutions are strictly adhering to what has been recommended. While teaching the core academic topics of “learning, memory, motivation, personality and intelligence” in the pre-clinical years in the early 80s at the Christian Medical College, Vellore, it was observed that the students did not evince much interest in learning the theoretical concepts of general psychology, since they were not examined in the topics taught. Moreover, the students could not relate the core subjects of general psychology to the clinical practice of medicine. However, when the same topics were dealt with during their clinical posting in psychiatry, there was more interest in learning about the different psychological tests that assessed the cognitive functions and their usefulness in diagnosing in different types of disorders, including mental retardation and dementia. Therefore, in order to achieve the learning objective of the curriculum, the psychology topics may have to be shifted to the clinical years, along with psychiatry, and may be duly examined. Majumdar *et al.*[3] reviewed the medical institutions in Southeast Asia, including India, and observed that colleges are experiencing difficulties in providing the right quality and quantity of educational experiences as the curricula have failed to respond to the needs of the community and the respective countries. There is also a need to include subjects on the behavioral responses to some of the “killer” diseases, including HIV and AIDS.

Moreover, from the perspective of a psychologist, the psychological component in different diseases, including stress in cardiology disorders and various other disorders, that has a bearing on behavioral changes has to be highlighted. Implementing basic behavioral principles for making the patients and relatives prepared for therapeutic adherence is a major challenge, and proper communication with this objective in mind may achieve better compliance. In the absence of a behavioral medicine specialist or a health psychologist as part of the health-care team, imparting skills on these lines may reduce the burden of patients and health costs.

## WHO SHOULD IMPART TRAINING?

The MCI does not specify which topic is to be taught by whom, but it recommends integrated training collaborating with “the departments of Medicine, Neuroanatomy, Behavioral Sciences and Forensic Medicine.” However, Prabhu pointed out as early as in 1969[4] and reiterated in 1997[5] that “In India, the person best suited to teach the subject is a qualified clinical psychologist.” Unlike in the last century, the training of clinical psychologists has undergone revision, and all the clinical psychology training centers that operate in the medical-college settings follow the curriculum that requires the clinical psychologist to work in different medical specialty settings, including pediatrics, neurology, cardiology or even nephrology. Hence the clinical psychology educators have the advantage in understanding and therapeutically responding to the psychological reactions of different medical conditions. That knowledge and skill imparted to the medical student is likely to make a great impact on the doctor-in-making for taking up future challenges.

## THE PSYCHOLOGICAL HELPING NEEDS OF THE MEDICAL STUDENTS

### In the initial phase

While conducting small group interactions with first year students in the initial months of their entry, they expressed different types of problems in relation to the studies. Inability to concentrate, inability to score high marks and difficulty in remembering new terms also came up. Those staying away from home expressed several difficulties. Apart from homesickness and problems of adjustment related to change in life styles and living conditions, some of them required individual attention since their perceptions were distorted. Though it may not be a significant problem for the urban and upper socioeconomic class students, medium of instruction is also an issue which the medical educators sometimes overlook. Students choose medical profession for various reasons, and some of them still take up the profession because of parental pressure. Even those who choose the subject of study on their own get disillusioned in the first few months of their entry to the medical school. Fainting at the cadaver table; fear about anatomy labs and frequent tests, and the poor scores they obtain in the initial days; which they were not used to in their premedical classes; which they were not used to all continue to be stressors for the new entrants. How the students are able to cope with the stressors sometimes depends on the type of institutions the students are in. It has been observed that personal attention to students definitely helps the process of learning, more so in disciplines where there is human relationship involved. Who would take care of these needs of the medical student is a question to be answered. Goudur and Kotur[6] opined that medical students are not receiving one-to-one attention at any stage of their education, which was once given prime importance. The cultural notion on education wherein the devout disciples learnt the art and science of healing, they feel, has been replaced with an “age of rolling out modern-day physicians along a conveyor belt.”

Some colleges adopted the practice of providing foster parent, wherein one of the senior faculty members is assigned a group of students who belong to different batches at different levels; the students of the group get an opportunity to meet the teacher informally, and the group interaction acts as a buffer, especially for the new entrant. The newcomer's informal discussions with the faculty member in the small-group setting, along with those who are studying in senior classes, help him/her allay apprehensions about the course and the new learning environment.

## PERSONAL ISSUES

When the students get an opportunity to interact with psychologists, some of them get back seeking help for either their own problems or those of the family members. The types of problems with which they present extend from career guidance to premarital counseling. The presence of a student counselor or a psychologist in medical institutions provides an opportunity for the students to get professional help. In Sri Ramachandra University at Chennai, when one of the clinical psychology faculty members served also as a student counselor of the institution, the type of problems that were handled, to list a few, included adjustment difficulties of the students to the teaching situation at different levels, disinterest to continue studies, difficulty in adjusting with teachers and peers, issues related to relationships, difficulty in making a career choice at the postgraduate level and problems related to premarital counseling. There were also situations where the medical students who required psychiatric help needed to be encouraged to take help and maintain therapeutic adherence. Sometimes the personality of the individual may not be matching with that required for practicing medicine. In such circumstances, career counseling that aims to help the undergraduate to choose a career that suits his/her personality might help the student to complete the undergraduate program successfully.

## DOCTOR-PATIENT RELATIONSHIP

Studies have shown that there is a great need for training in the aspect of doctor-patient relationship. Obtaining informed consent of the patient or relatives, breaking unpleasant news to the significant relatives, informing about critical illnesses to patients, all require good amount of communication skills. It is a complex task, to be performed at critical moments when there are lot of uncertainties and when decision has to be taken without having opportunity and time to discuss with a colleague. Patient-doctor relationship itself has an important bearing on the healing process and better patient compliance. Its importance has become highly relevant with increasing awareness about consumer and human rights among patients and relatives, which is likely to pose a threat to the doctor, and a proper training could ease stress of the doctor-in-making.

Worley,[7] analyzing community-based medical education, has delineated four types of relationships that are needed in undergraduate medical education. Apart from the clinical, the doctor-patient relationship, he highlights the importance of social, community and interpersonal relationships that are needed for community-based medical education. With the *Panchayat Raj* system being introduced in the country, for the doctors working in primary health center, as well as those working in other health centers, the society lays a great demand on the doctors' skills with regard to interpersonal relationship, at various local administrative forums. Therefore, the content on formation and maintenance of healthy relationships and interpersonal communication needs to be included in the curriculum.

Clinical empathy, which involves the ability to understand the patient's situation, perspective and feelings; and the ability to communicate with the patient in an accurate and effective manner have a major role to play in patient care. Mercer and Reynolds[8] emphasized that empathy can be enhanced and successfully imparted in medical schools, provided it is embedded with the actual experiences of students with the patients.

With the inclusion of psychiatric care in primary-care centers, the primary-care physician is expected to identify, treat or refer the major psychiatric disorders, various disabilities, including intellectual disability and prevent some of the disorders. The attained skills in relating would help in fulfilling these objectives. Tharyan *et al.,*[9] in their study, observed that exposure to psychiatry training did have an influence on the change in attitude to mental illness, though it did not influence the choice of career. Rao *et al.*[10] did a prospective study to elicit and monitor over time the medical students' attitudes to psychiatry, psychiatrists, psychiatric patients and their treatment. There was a significant shift in favorable direction in the general attitude to psychiatry, both before and after training. Majority of them considered psychiatric specialty as challenging and scientific, and almost all felt that too little time is being spent on psychiatry in UG curriculum. Another study on the same subjects[11] to know their interest to specialize in psychiatry provided very interesting findings before and following training at UG level. Sixteen percent of boys were sure, 28% undecided and 56% against a career in psychiatry; while no female student considered career in psychiatry, but 50% were not sure. Majority of students had opted for medical and surgical branches. Interest in another branch of medicine was the commonest explanation given for not intending to take up psychiatric specialization. The conclusion reached in the editorial published in the Lancet[12] on “Who puts medical students off psychiatry?” was that the best teaching in the world is unlikely to prevail against the poor working conditions, a bad professional image and the frustrations of dealing with society's misfits and people who rarely appeared cured; and this may be equally true even today in the Indian context. Even though the above statement may look pessimistic the bright spot is that almost all of our students mention “too little time devoted to psychiatry in medical curriculum”; which needs immediate correction, in addition to improvement in teaching methods. Another related neglected area is sexuality training, which is very close to psychiatry.[13] Evidence has been provided for the need to improve knowledge about different aspects of sex among a sample of Indian medical students.[14]

## REFLECTIVE PRACTICE IN MEDICINE: A NEW APPROACH

Goudar and Kotur,[6] while reviewing trends in medical education in the country, made a very strong statement – “Most faculty who teach are unaware of the literature on cognitive psychology, adult learning and the development of mastery and expertise.” Their observations have to be taken seriously when one looks at the innovations that came into the teaching methods in different disciplines, including that of medicine. With the use of computer-assisted instruction, simulated practices; and the application of closed-circuit observational methods to telemedicine, there has been tremendous revolution in teaching-learning process based on evidence. A comprehensive review of medical education technology has been done by Bhuiyan and Rege[15] in their article on Evolution of Medical Education Technology Unit in India. For the development of expertise in professional practice, the capability to reflect consciously upon one's practice is considered important. Schmidt and Rikers[16] focused on the question of how knowledge is organized in the doctor's mind; and conceptualized that development of encapsulated knowledge, followed by the formation of illness scripts, may both be considered as important stages in the development of medical expertise. Mamede and Schmidt[17] opined that training students to apply reflective practices may eventually become the goal in medical education.

Radhakrishnan,[18] the great educator of our country, suggested that the teacher should initiate the students into “… a life of spirit that has been our ideal for centuries.” He reiterated that educators should be conscious that we in India live in the midst of spiritual world that dominates over the material, and the reflective practices should help the student to integrate with the sociocultural milieu.

## NEED FOR REVISING THE SELECTION PROCEDURE

Currently the selection of the student into the undergraduate course is through an entrance test, conducted at the all-India level, state level or at the institution in some of the notified colleges and private institutions, for those who fulfill the eligibility criteria based on the marks achieved in the qualifying examination specified by the MCI.[2] The students have the wider option of taking the entrance examination of different private medical colleges to which they chose to apply. The entrance examination of majority of the medical institutions assesses only the academic ability in the cognitive domain. As an exception, some institutions follow a different approach, wherein the prospective students are provided with an opportunity to bring out the best potential in interpersonal skills, commitment, aptitude and personality traits that are suitable for the profession. Though this method is a highly effective one, the procedure takes long time and involves good training on the part of the selectors, as is done in the assessment centers of the military. Candidates numbering more than double the number of available seats are called in for an interview and thorough observation of each candidate in different tasks, both individual and group situations; group discussion; and lengthy serial one-to-one in-depth interviews that help to evaluate the positive and the negative aspects of the prospective candidate. At the national level or at the state level, it will be difficult to adopt this practice due to the wide range of differences that exist across the country and the possibility of bias that could creep in. However, there is a need to screen the students with respect to their interpersonal skills and emotional responses that are closely linked with the profession. One of the alternatives is to include Situational Judgment Tests (SJT) that present the applicants with job-related situations and possible responses to the situations.[19] These would help select candidates who are likely to excel in the profession.

In summary, there is a need to revise the curriculum and to specify the method by which psychology topics are taught in a practically oriented manner, so that the physician could apply the concepts in clinical practice. The method of teaching medical subjects may be modified in order to give adequate room for reflective practice. The selection procedure may be revamped to select suitable candidates with the profession-related skills, in addition to screening in the cognitive domain.
